# A Ser75-to-Asp phospho-mimicking mutation in Src accelerates ageing-related loss of retinal ganglion cells in mice

**DOI:** 10.1038/s41598-017-16872-7

**Published:** 2017-12-01

**Authors:** Kenji Kashiwagi, Sadahiro Ito, Shuichiro Maeda, Goro Kato

**Affiliations:** 1 Department of Ophthalmology, University of Yamanashi, Yamanashi, 409-3898 Japan; 2 Department of Biochemistry, Faculty of Medicine, Graduate Faculty of Interdisciplinary Research, University of Yamanashi, Yamanashi, 409-3898 Japan; 3 Present Address: Center for Life Science Research, University of Yamanashi, 1110 Shimokato, Chuo, Yamanashi, 409-3898 Japan

## Abstract

Src knockout mice show no detectable abnormalities in central nervous system (CNS) post-mitotic neurons, likely reflecting functional compensation by other Src family kinases. Cdk1- or Cdk5-dependent Ser75 phosphorylation in the amino-terminal Unique domain of Src, which shares no homology with other Src family kinases, regulates the stability of active Src. To clarify the roles of Src Ser75 phosphorylation in CNS neurons, we established two types of mutant mice with mutations in Src: phospho-mimicking Ser75Asp (SD) and non-phosphorylatable Ser75Ala (SA). In ageing SD/SD mice, retinal ganglion cell (RGC) number in whole retinas was significantly lower than that in young SD/SD mice in the absence of inflammation and elevated intraocular pressure, resembling the pathogenesis of progressive optic neuropathy. By contrast, SA/SA mice and wild-type (WT) mice exhibited no age-related RGC loss. The age-related retinal RGC number reduction was greater in the peripheral rather than the mid-peripheral region of the retina in SD/SD mice. Furthermore, Rho-associated kinase activity in whole retinas of ageing SD/SD mice was significantly higher than that in young SD/SD mice. These results suggest that Src regulates RGC survival during ageing in a manner that depends on Ser75 phosphorylation.

## Introduction

Src, a membrane-associated 60 kDa tyrosine kinase, is expressed ubiquitously in mammalian tissues and is involved in the regulation of growth and post-mitotic cell behaviour^1–3^. The activation of Src occurs during fibroblast mitosis and is accompanied by the phosphorylation of serine and threonine in the amino-terminal Unique domain of the protein^4^. Cyclin-dependent kinase 1 (Cdk1; also known as p34^cdc2^), a critical cell-cycle regulator activated at the onset of mitosis, phosphorylates these mitosis-specific phosphorylation sites of Src^5,6^. Src functions in the regulation of mitosis by transducing Cdk1-initiated signals through phosphorylation cascades^7^. Cdk1-mediated phosphorylation of Src controls its mitotic activation that is modulated by Tyr527 phosphorylation^8,9^.

Although neurons are post-mitotic, they express high levels of Src, and a neuronal form of Src is expressed in some neural tissues of the brain and retina^10,11^. Src-specific activity is higher in neurons than in non-neuronal cells, suggesting that the protein plays important roles in neurons. For example, Src is involved in neurite extension, *N*-methyl-D-aspartate receptor-mediated synaptic transmission, and plasticity^2,12^, in addition to pathological processes, such as neurodegeneration, in the central nervous system (CNS)^13^. Nevertheless, Src knockout mice do not exhibit detectable abnormalities in neural tissues, although these mice do have shorter lifespans and develop osteopetrosis^14,15^. To date, however, studies of these animals have not yielded a full understanding of the physiological role of Src^14^, and it is possible that the observations in these knockout mice may reflect functional compensation by other tyrosine kinases related to Src^15^. Thus, to address this issue, we postulated that it would be useful to introduce point mutations into *src* at sites associated with the regulation of specific functions of Src.

One of the mitotic phosphorylation sites in human SRC is Ser75, located in the Unique domain of the protein. In human retinoblastoma cells, Ser75 is phosphorylated in a mitosis-independent manner^16^. This phosphorylation occurs in cultured neurons and some cultured tumour cells expressing neuronal forms of Src^17^. Previously, we showed using retinoblastoma cells that the kinase Cdk5/p35, which has the same consensus sequence as Cdk1, is responsible for phosphorylating Ser75 in the Unique domain, suggesting that Ser75 phosphorylation may play important roles in CNS neurons^16,18^.

Therefore, to gain a greater understanding of the role of Src in CNS neurons, we established two mutant mouse lines expressing one type of mutant Src each: one with a Ser75-to-Asp (SD) mutation, mimicking the phosphorylated form, and the other with a Ser75-to-Ala (SA) mutation, which lacks the phosphorylation site. Because these alleles harbour point mutations in the Unique domain, which shares no sequence similarity with other *src* family kinases, these mice were predicted to exhibit detectable abnormalities.

## Results

### Generation of SD and SA mutant mice

We established two lines of mice, each expressing one Src mutant: the phospho-mimicking Src S75D (SD) and the non-phosphorylatable Src S75A (SA). SD and SA mutant mice carried TCC → GAC and TCC → GCG mutations, respectively, in codon Ser75 of the mouse c-*src* gene.

**Figure 1:**
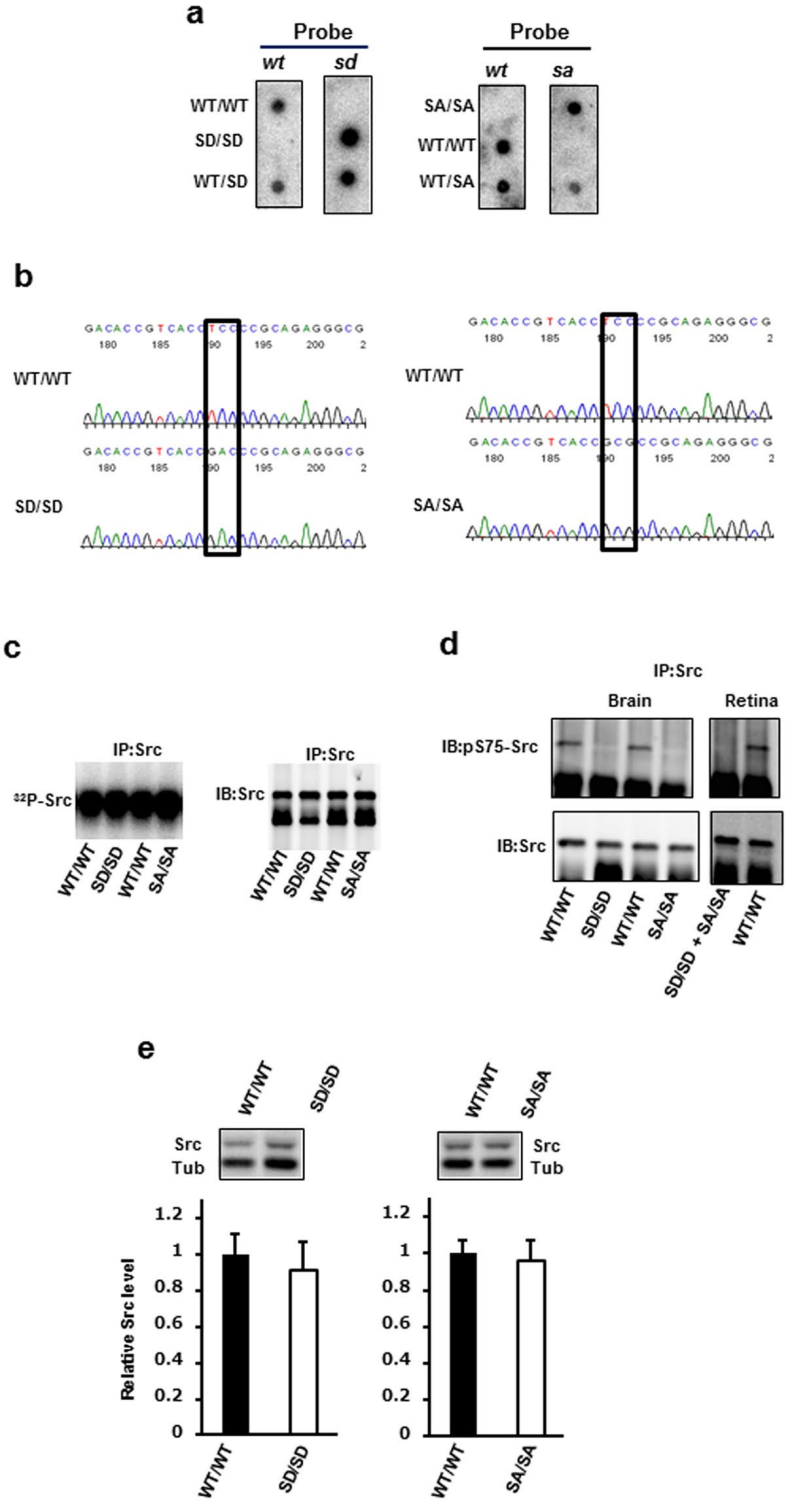
Generation of Src S75D (SD) and Src S75A (SA) mutant mice and verification of mutations. (**a**) Genotype analysis of littermates derived from SD or SA heterozygotes. Tail DNA was amplified by PCR and subjected to dot-blot analysis with ASO probes. ASO probes *wt*, *sd* and *sa* were identical to the normal, SD mutant and SA mutant sequences, respectively. (**b**) Chromatogram showing the presence of the TCC → GAC and TCC → GCG mutations at codon Ser75 in SD/SD and SA/SA mutant mice, respectively. Src cDNA was reverse-transcribed from RNA isolated from retinas from mice of the indicated genotype, amplified by PCR and then subjected to sequencing. (**c**) Autophosphorylation kinase assay from neural tissue extracts. Brain lysates from WT/WT, SD/SD and SA/SA mice aged 6 months were immunoprecipitated with monoclonal anti-Src antibody 327 and then subjected to immunoblot analysis (right) and autophosphorylation assay (left). Protein images were acquired using a LAS 1000 Image Analyzer. Autophosphorylation was detected using a BAS 2500 Image Analyzer. IP, immunoprecipitation; IB, immunoblot. (**d**) Lack of Ser75 phosphorylation in Src from the mutants. Brain (left) or retina (right) lysates from WT/WT and mutant mice aged 17–22 months were immunoprecipitated with anti-Src antibody 327 and subjected to immunoblot analysis with an anti-Src phospho-Ser75 antibody. Blots were stripped and reprobed with anti-Src antibody 327. Equal amounts of retinal lysates from SD/SD and SA/SA were mixed and analysed as described above. (**e**) Quantitation of Src protein levels in the retina. Cell lysates were prepared from the retinas of SD/SD, SA/SA and their WT counterparts aged 6–8 months and then subjected to anti-Src immunoblot analysis. Src protein levels were normalised to those of 55 kDa β-tubulin (Tub). n = 8 for WT/WT and SD/SD; n = 7 for SA/SA. Data are means ± s.d. Unpaired two-tailed *t*-test. The full-size dot blots for Fig. 1a, the full-length autoradiograph for Fig. 1c, and the full-length blots for Fig. 1d,e are presented in Supplementary Figs S2–S5.

To analyse the phenotypes of SD/SD and SA/SA mice, we intercrossed their respective heterozygotes. Tail DNA was amplified by polymerase chain reaction (PCR), and then allele-specific oligonucleotide (ASO) probe hybridisation was performed to determine the genotypes of the SD or SA littermates (Fig. 1a). To confirm transcription of the SD and SA mutant alleles, we isolated c-*src* cDNA by reverse transcription-PCR from wild-type (WT)/WT and homozygous mutant littermates. The mutations were verified by sequencing (Fig. 1b). The entire Src open reading frame was sequenced to confirm that no other mutations were present.

To examine Src kinase activity in SD and SA mutant mice, we performed autophosphorylation assays using immunoprecipitated Src from whole brain extracts of WT/WT and homozygous mutant mice. Like WT/WT mice, SD/SD and SA/SA mice expressed active Src (Fig. 1c). To confirm that phosphorylation was not occurring in SD and SA mutant mice, we performed immunoblots using an anti-Src (phospho-Ser75) antibody to detect Ser75 phosphorylation in brain or retina lysates following immunoprecipitation with the anti-Src antibody. Ser75 was phosphorylated in Src derived from WT/WT, but not in Src obtained from SD/SD or SA/SA mice (Fig. 1d).

To ensure that genetically altered and WT mice expressed the same level of Src protein, we assayed the retinal Src protein level by immunoblot analysis in the mice aged 6–8 months (Fig. 1e). No significant difference in the retinal Src protein level was detected between SD/SD or SA/SA mice and their WT counterparts. These results indicate that any changes observed in the SD/SD or SA/SA mutant mouse retina must be attributed to the SD or SA mutation itself, rather than to altered levels of Src protein.

Mice heterozygous or homozygous for the SD and SA mutations were viable, fertile and born at the expected Mendelian ratio. They exhibited no obvious abnormalities in appearance, and appeared to have a normal lifespan relative to their WT/WT littermates.

### Retinal ganglion cell (RGC) loss in ageing SD/SD mutant mice

**Figure 2:**
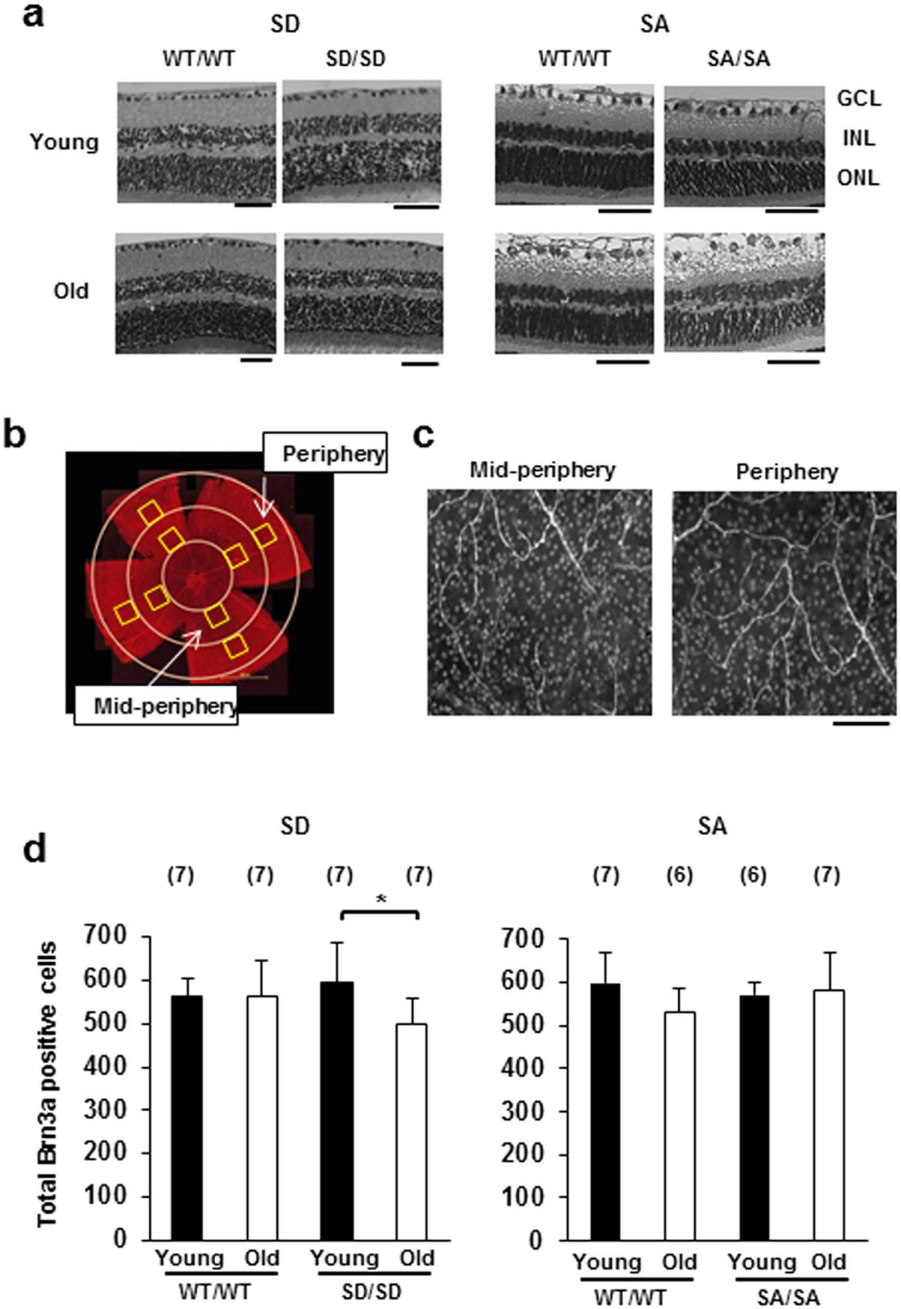
RGC loss in aged SD/SD mutant mice. (**a**) Histological examination of SD/SD (left) and SA/SA (right) mutant mid-peripheral retinas stained with haematoxylin and eosin. Retinal sections were prepared from young (3 months old) and ageing (18 months old) SD/SD and control WT/WT mice, and from young (5 months old) and ageing (20–21 months old) SA/SA and control WT/WT mice. Young, young mice; Old, ageing mice; GCL, ganglion cell layer; INL, inner nuclear layer; ONL, outer nuclear layer. Scale bars, 50 μm. (**b**) Schematic diagram of the locations of mid-peripheral and peripheral regions in the retina immunostained with Brn3a antibodies. Whole retinas were immunostained with a mouse anti-Brn3a monoclonal antibody. Scale bar, 1,000 μm. (**c**) The numbers of Brn3a-positive RGCs were determined in the four boxed areas of equal size (400 × 400 μm) in the mid-peripheral and peripheral regions. Representative images of young WT/WT mice (control of SD/SD mice) are shown. Scale bar, 50 μm. (**d**) Quantitation of RGC number in whole retinas from SD/SD (left) and SA/SA (right) mutant mice. Brn3a-positive RGCs were counted in the whole retinas, in which mid-peripheral and peripheral regions were combined. Sample numbers are shown in parentheses. Young, young mice; Old, ageing mice. Data are means ± s.d. Sample numbers are shown in parentheses. Factorial ANOVA and Bonferroni post hoc test, *P < 0.05.

To examine the effect of the SD and SA mutations on retinal tissue, we performed histological analyses of neural retinas obtained from SD/SD and SA/SA mutant mice at younger (3–6 months old) and older (16–23 months old) ages. No structural abnormality was observed in the retinas of young and ageing SD/SD (Fig. 2a, left) and SA/SA mutants (Fig. 2a, right), as well as no inflammatory cell infiltration, neovascularisation, oedema or haemorrhage.

The Brn3a protein, a member of the Brn3 family of Pit-Oct-Unc-domain transcription factors, is specifically expressed by retinal ganglion cells (RGCs) in mice. Thus, Brn3a is a reliable protein marker for the identification and quantitation of RGCs in whole retina^19–21^. To investigate RGC loss, we quantitatively analysed Brn3a-positive RGC numbers in the mid-peripheral and peripheral regions of whole retinas (Fig. 2b,c) obtained from young and ageing mutant mice and compared these values with those obtained in young and ageing WT mice.

The RGC number of the whole region (mid-peripheral and peripheral regions combined) was reduced by 16% (*P* = 0.0171) in ageing SD/SD mice relative to that in young SD/SD mice, whereas WT/WT mice exhibited no age-related change in RGC number (Fig. 2d, left). By contrast, the RGC number in the retina of SA/SA mice was unchanged by age (Fig. 2d, right).

### ROCK activity upregulation during ageing

**Figure 3:**
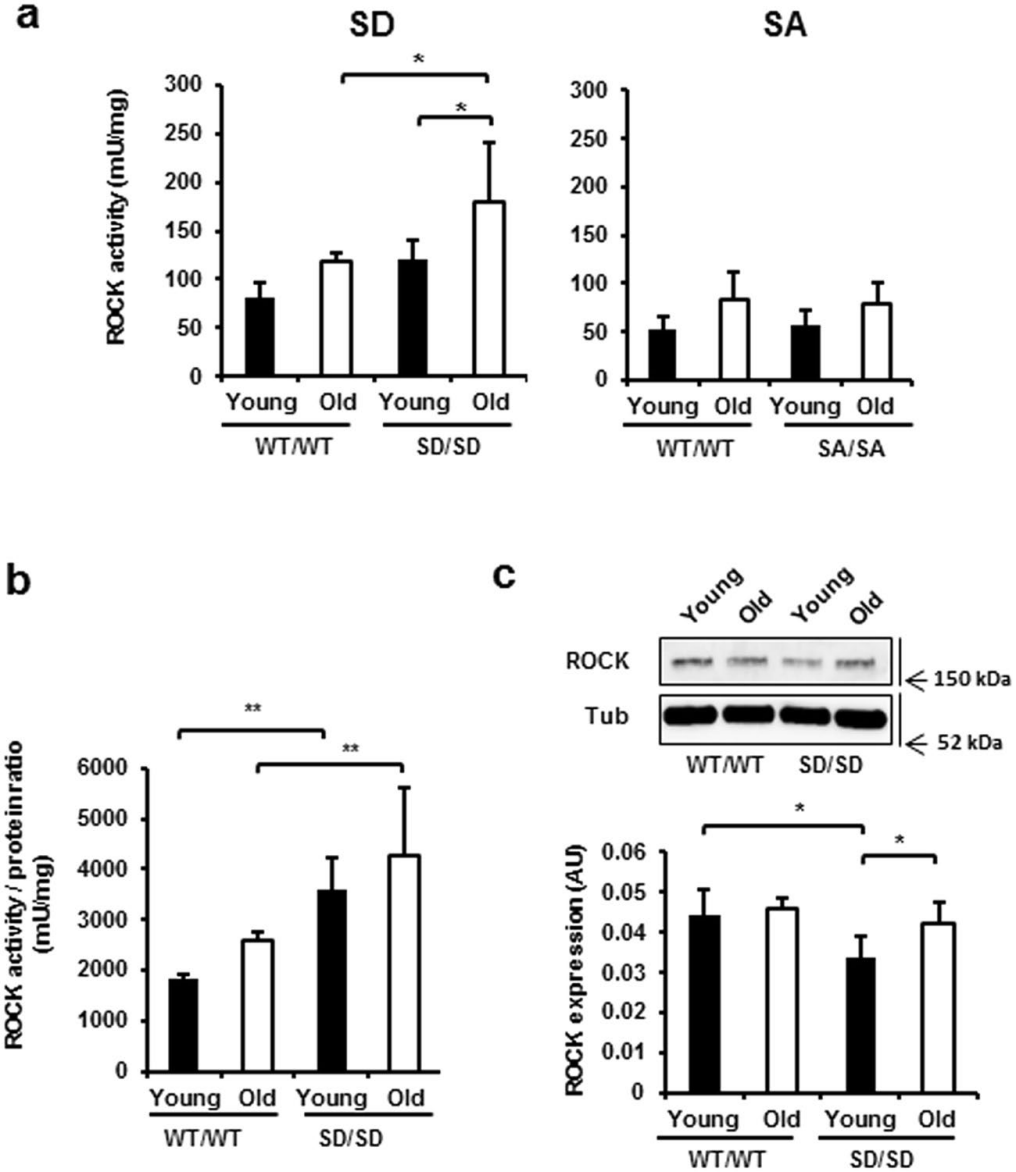
Age-related upregulation of ROCK in retinas of SD/SD mutants. (**a**) ROCK activity levels in the retinas of young and ageing SD/SD (left) and SA/SA (right) mutant mice. A whole retina isolated from each mouse eye was lysed, and the lysate (3 μg) was subjected to immunoassay for ROCK activity. (**b**,**c**) ROCK activity/protein ratios. The lysate of each retina (10 μg) was subjected to anti-ROCK immunoblot analysis (**c**). ROCK protein levels were normalised to those of 55 kDa β-tubulin (Tub) and used to estimate the ROCK ratios (**b**). Young, young mice; Old, ageing mice. n = 4. Data are means ± s.d. Factorial ANOVA and Bonferroni post hoc test, *P < 0.05, **P < 0.01. The full-length blot for Fig. 3c is presented in Supplementary Fig. S6.

The RhoA/Rho-associated kinase pathway plays roles in age-related neurodegeneration^22,23^. To elucidate the mechanism by which the SD mutation affects age-dependent RGC loss in whole retina, we analysed ROCK activity in whole retinas obtained from young and ageing WT/WT and SD/SD mice (Fig. 3a, left). ROCK activity in WT/WT did not change with age (*P* = 0.1323). By contrast, ROCK activity in whole retinas from ageing SD/SD mice was higher than that in both young SD/SD (48%; *P* = 0.0257) and ageing WT/WT mice (51%; *P* = 0.0216). These ROCK activity changes were inversely correlated with RGC number in whole retinas. The levels of ROCK activity in whole retinas obtained from SA/SA and WT/WT mice were similar at young and older ages, and the activity did not increase with age in SA/SA mice (*P* = 0.1736; Fig. 3a, right).

Because ROCK is a downstream effector of the Src-dependent phosphorylation cascade^24^, we investigated whether the increased ROCK activity observed in whole retinas obtained from SD/SD mutant mice was due to activation of ROCK kinase activity or to increases in the ROCK protein level (Fig. 3b,c). ROCK activity/protein ratios were compared between young and ageing WT/WT and SD/SD mice. The ratio in the retina was higher in young and ageing SD/SD mice than in young and aging WT mice (97%; *P* = 0.0055 and 65%; *P* = 0.0075, respectively). The ROCK ratio was not significantly affected by the age of either WT/WT or SD/SD mice (Fig. 3b). On the other hand, the ROCK ratio in retinas obtained from young SA/SA mice was similar to that in young WT/WT mice, suggesting that the SA/SA mutation did not activate ROCK kinase activity in whole retina (Supplementary Fig. S1, left). In contrast with these results, the level of retinal ROCK protein in young SD/SD, but not SA/SA (Supplementary Fig. S1, right) mutant mice was lower than that in young WT/WT mice (24%; *P* = 0.0105; Fig. 3c). The ROCK level increased with age in SD/SD mice (24%; *P* = 0.0355), whereas age did not affect the ROCK level in WT/WT mice (Fig. 3c). Thus, the SD/SD mutation increased ROCK kinase activity and was associated with an age-related increase in the ROCK protein level.

### RGC losses in the mid-peripheral and peripheral regions of the retina

**Figure 4:**
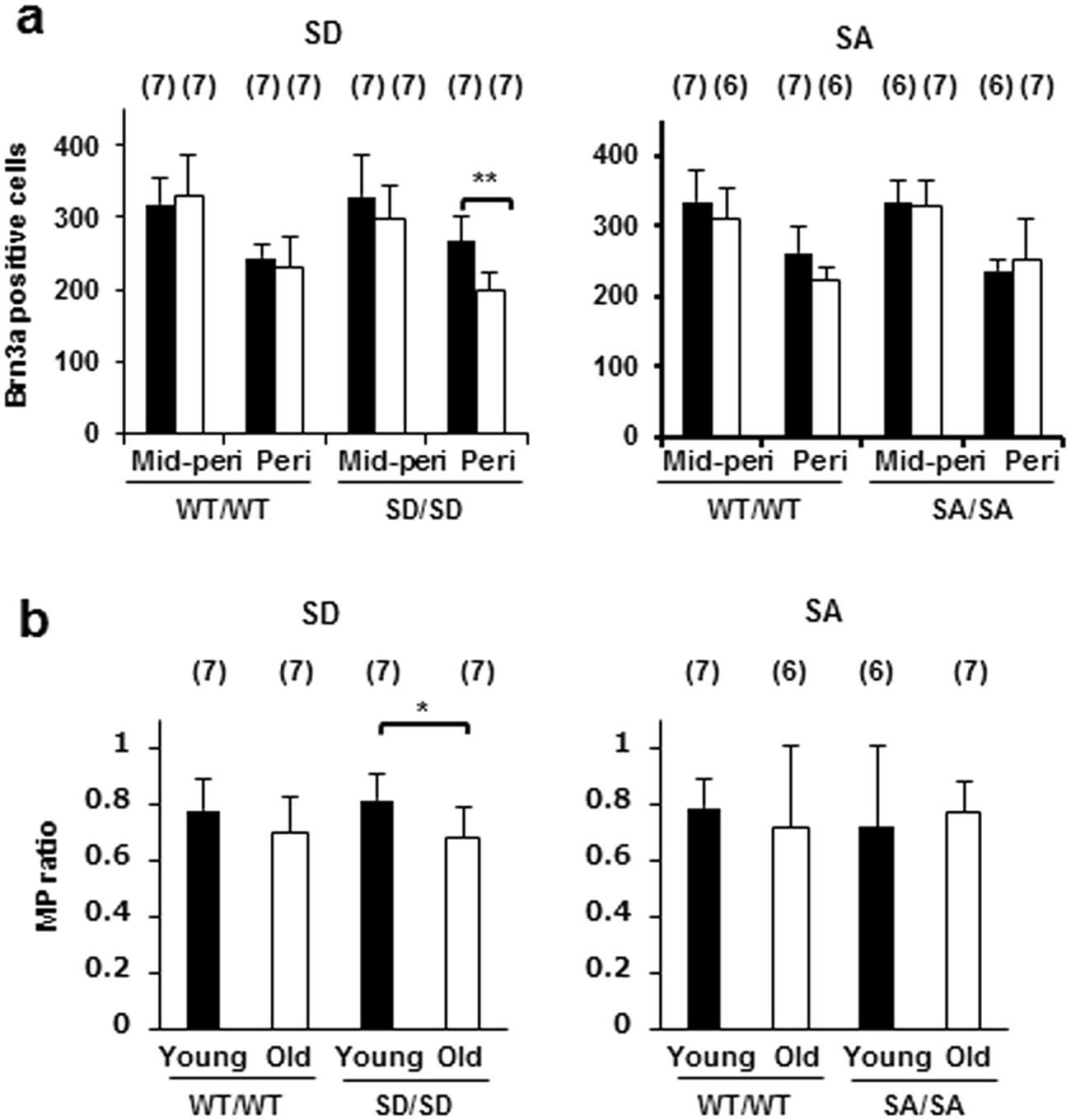
RGC numbers in mid-peripheral and peripheral regions of retinas derived from SD/SD and SA/SA mutant mice. (**a**) Brn3a-positive RGCs were counted in mid-peripheral and peripheral regions of retinas obtained from young (■) and ageing (□) SD/SD (left) and SA/SA (right) mutant mice. Paired factorial ANOVA and Bonferroni post hoc test, **P < 0.01. (**b**) Ratios of RGC number in the retina at the periphery to that at the mid-periphery. The ratios (MP ratio) were calculated for WT and homozygous young and ageing SD/SD (left) and SA/SA (right) mutant mice. Factorial ANOVA and Bonferroni post hoc test, *P < 0.05. (**a**,**b**) Mid-peri, mid-peripheral region; Peri, peripheral region; Young, young mice; Old, ageing mice. Sample numbers are shown in parentheses. Data are means ± s.d.

We compared the age-dependent RGC loss in the mid-peripheral and peripheral regions of the retina among the genotypes. Although no significant age-related change was observed in the number of RGCs in the mid-peripheral and peripheral regions of retinas obtained from WT/WT mice (*P* = 1.0000 and 1.0000; Fig. 4a, left), the retinas derived from the SD/SD mice exhibited a significant age-related decrease of 25% (*P* = 0.0008; Fig. 4a, left) in the number of RGCs in the peripheral but not in the mid-peripheral region (*P* = 0.3712; Fig. 4a, left). By contrast, ageing SA/SA mice exhibited no significant age-related change in RGC number in the mid-peripheral and peripheral regions relative to young SA/SA mice (*P* = 1.0000 and 1.0000; Fig. 4a, right); and their ageing WT counterparts exhibited no significant age-related change in RGC number in the mid-peripheral and peripheral regions (*P* = 0.6951 and 0.0644; Fig. 4a, right).

The number of RGCs in the peripheral region of the retinas in both young and ageing mice was lower than that in the mid-peripheral region of all genotypes (*P* < 0.0001; Fig. 4a). The ratios of the numbers of RGCs in the peripheral region relative to those in the mid-peripheral region (MP ratio) were compared between young and ageing mice. Only in SD/SD mice was the MP ratio in ageing animals significantly lower (17%) than that in young animals (*P* = 0.029; Fig. 4b, left). Consistent with this result, in SD/SD mice, the ratio of average RGC numbers in aged relative to young mice (young:old ratio) in the peripheral region (0.75) was 18% lower than that in the mid-peripheral region (0.91) (Fig. 4a, left). By contrast, no age-related change was observed for the MP ratio in SA/SA mice (Fig. 4b, right). The young:old ratio of average RGC numbers in the peripheral region (1.07) of SA/SA mice was similar to that in the mid-peripheral region (0.98) (Fig. 4a, right).

These results suggest that the RGCs in the peripheral region rather than those in the mid-peripheral region of the retina are preferentially damaged during ageing in SD/SD mice. By contrast, the SA/SA mutation did not promote RGC loss in the peripheral retina during ageing.

### Intraocular pressure (IOP) elevation

**Figure 5:**
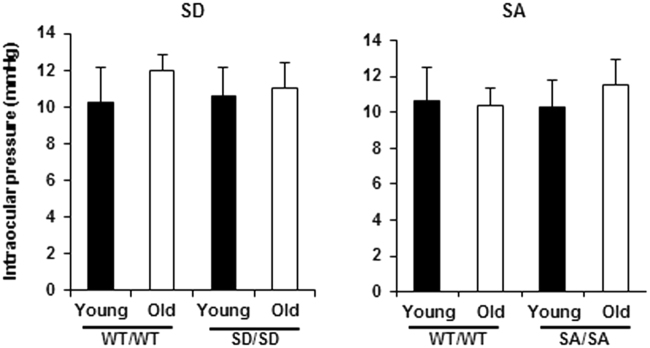
IOP measurements in young (3–6 months) and ageing (16–18 months) SD/SD and SA/SA mutant mice. IOP was measured with a rebound tonometer. n = 4. Data are means ± s.d., Factorial ANOVA and Bonferroni post hoc test. Young, young mice; Old, ageing mice.

Higher intraocular pressure (IOP) is considered to be the most important risk factor for the onset and deterioration of glaucoma. We, therefore, measured IOP in young and ageing mice of all three genotypes to investigate the relationship between RGC loss and IOP elevation in this model. To evaluate the relationship between RGC loss and IOP elevation, we measured IOP in young and ageing mice of all three genotypes (Fig. 5). We found no age-related difference in the IOP in either SD/SD or WT/WT mice (Fig. 5, left), indicating that the RGC loss in the SD mutant mouse was independent of IOP elevation. The IOP in ageing SA/SA mice was unchanged relative to that in young SA/SA mice and their WT counterparts (Fig. 5, right).

## Discussion

This *in vivo* study demonstrated that the phospho-mimicking mutant Src S75D, but not the non-phosphorylatable mutant Src S75A, was associated with increased RGC loss in the retina, especially in the peripheral region, without an elevation in IOP, in ageing mice, suggesting that Ser75 phosphorylation regulates RGC survival during ageing. This age-related RGC loss was inversely related to ROCK activity in the whole retina.

In the neural retina, Src is expressed mainly in RGCs and photoreceptor cells, but its function remains obscure^11,25^. Because a good *in vivo* model to directly test the specific roles of Src while excluding the effects of redundant *src* family kinases is lacking^15^, the roles of Src in the RGC remain unknown. Ser75 in the Unique region of the *src* gene does not exist in other Src family kinase members and is highly conserved in Src, even in phylogenetically distant species. Our mutant mice carrying point mutations at Ser75 may overcome the challenge related to this redundancy. Moreover, unlike Src-null mutant mice^14^, the mutant mice developed here appeared to possess a normal lifespan and morphology. Thus, we believe that our mutant mice, carrying point mutations at Ser75, represent useful animal models for investigating the physiological roles of Src Ser75 phosphorylation in RGC survival.

The current mutant model showed age-related RGC loss without any morphological damage to other retinal components (Fig. 2a). Moreover, the IOPs of the mutants did not exceed those of their WT counterparts (Fig. 5). Taken together, these results suggest that the mutant mice could provide useful *in vivo* and *in vitro* systems for investigating the pathogenesis of age-related neurodegenerative diseases, such as normal tension glaucoma^26^, and developing therapies against these diseases.

Recent evidence suggests that downregulation of the small GTPase RhoA promotes survival and regeneration of RGC^27^. Inhibition of ROCK, one of the major downstream effectors of RhoA, increases neurite outgrowth and supports RGC survival^28–32^. In the present study, we showed that age-related RGC loss in SD/SD mice was inversely correlated with an age-related increase in the activity of ROCK, a downstream effector of Src, in the whole retina. Despite the fact that the retinas of young SD/SD mice contained approximately a 2-fold higher ROCK activity/ROCK protein ratio than WT mice, retinal ROCK activity in young SD/SD mice was not significantly different from that in young WT mice. This is because the ROCK protein level was lower in the retinas of young SD/SD mice, unlike the retinas of SA/SA mice (Fig. 3 and Supplementary Fig. S1). In young mice, ROCK protein levels may be downregulated in response to the increase in the ROCK ratio. However, with ageing, downregulation may decline, resulting in high ROCK activity and significant RGC loss in the retinas of ageing SD/SD mice. These results suggest that the SD mutation and ageing together affect RGC survival; that is, the SD gene mutation in these mice predisposes them to age-dependent RGC loss.

Some evidence has been obtained regarding the biochemical functions of Ser75 phosphorylation in the Src Unique domain^33–35^. In cultured human lens epithelial cells, ubiquitin-mediated degradation of activated Src is accelerated by Cdk5-dependent phosphorylation of Src Ser75^36^. This Cdk5-dependent suppression of active Src inhibits ROCK activity and results in cytoskeletal contraction and cell migration in lens epithelial cells^37^. Cdk5/p35 and ROCK are associated with survival and cytoskeletal activity of RGCs *in vitro* ^38^. Two possible pathways of Src-mediated downregulation of ROCK are proposed: one involves inactivation of RhoA through Src-dependent activation of p190RhoGAP^37,39^ and the other involves Src-dependent phosphorylation of ROCK^40^. Although the details of the molecular mechanisms remain to be clarified, the Src Ser75 mutation could affect fine-tuning of intracellular activity during ageing, resulting in deregulation of RGC survival and regeneration through ROCK.

We presented evidence for a difference in susceptibility to RGC loss in the mid-peripheral and peripheral retina: the RGCs in the periphery were preferentially reduced during ageing in SD/SD mutant mice. By contrast, the peripheral RGC loss was preferentially suppressed during ageing in SA/SA mutant mice. The mechanism is not known for this age-related difference in RGC loss susceptibility between the mid-peripheral and peripheral retina. The conditions to which the RGCs are exposed in the mid-periphery and periphery may differ; for example, the presence of glial cells^41,42^, expression of neurotrophic factors and their receptors^43,44^, redox status^43,45,46^, and axonal transport^47,48^ may differ. Future proteomics analyses of different regions of retina would lead to a better understanding of the mechanism^49,50^. In addition, specific ROCK activity or protein levels or both could be changed in relation to changes in Src Ser75 phosphorylation levels in the different regions of the retina.

Src Ser75 phosphorylation is a marker of the mitotic phase of the cell cycle. The G2/M regulator Cdk1 and its regulatory subunit cyclin B1 have been identified immunohistochemically in RGCs^51^. Aberrant expression or activation of the mitotic kinase in degenerating neurons has been investigated in studies aimed at understanding the mechanisms that induce cell death of post-mitotic neurons^52–55^. A large body of evidence implicates Cdk5 kinase in neurodegenerative diseases^56–59^. Expression of Cdk5 and its activator p35 changes over the course of development: Cdk5 expression is sustained in adulthood in both the inner nuclear layer and ganglion cell layer of the retina, but p35 expression is only sustained in the ganglion cell layer^60^. Furthermore, prolonged hyperactivation of Cdk5 has been inferred from Ca^2+^-activated, calpain-induced conversion of p35 to p25 in an acute glaucoma model^61^. Although some data exist suggesting the involvement of Cdk5 or Cdk1 kinase in retinal dysfunction^51,53,60,61^, their target molecules remain unknown. The results of the present study suggest that Src may be a candidate target molecule of active Cdk5 or Cdk1 during age-dependent neurodegeneration *in vivo*.

In conclusion, the phospho-mimicking SD mutant mice revealed that Src Ser75 phosphorylation regulates ageing-related RGC loss and that this is associated with altered ROCK kinase activity.

## Methods

### Animal experiments

Animal experiments were approved by the Animal Care and Use Committee of the University of Yamanashi and conducted in accordance with the University of Yamanashi’s Guide for Animal Experimentation and the ARVO Statement for the Use of Animals in Ophthalmic and Vision Research. Analyses were performed after the SD and SA mutant mice were backcrossed six times to C57BL/6.

### Generation of Mice

Knockin mice were produced using our previously reported gene-targeting procedure for the introduction of a point mutation into one allele of the c-*src* gene in mouse CCE embryonic stem (ES) cells by replacement and excision steps^62,63^. This procedure is feasible for generating mutant mice harbouring no exogenous sequence that may affect the expression of the targeted locus and closely linked genes.

The targeting vector was constructed as previously described^63^. It contained 9.0 kb of mouse c-*src* sequence, a 3.4 kb HSV-tk-neo cassette flanked by a 3.2 kb duplication containing a part of the c-*src* sequence. In the mutant constructs, exon 2 contained the 2 bp SD mutation (TCC→GAC) or the 2 bp SA mutation (TCC→GCG).

In our method, the replacement step involves homologous recombination between the targeting vector and the endogenous gene; this homologous recombination creates the mutation and inserts the selection cassette flanked by the duplication in one allele. After transfection of CCE ES cells with this vector, G418-resistant clones were selected and screened for homologous recombination by Southern blot analysis of genomic DNA^63^. The DNA was amplified by PCR^63^, and ASO probes specific for the normal or SD mutant allele were dot-blot hybridised to the PCR products to confirm heterozygosity, as described previously^63^. To detect the SA mutant allele, the PCR products were dot-blot hybridised at 54 °C with the ASO probe *sa*, which is identical to the SA mutant sequence (5′-TCTGCGGCGCGGTGAC-3′). The excision step excised the selection cassette by homologous recombination within the duplication at one allele. Heterozygous revertants bearing only the point mutation with no selection cassette were selected^63^ and screened for the excision by Southern blot analysis. ASO probe dot-blot hybridisation analyses were performed to confirm the presence of WT and mutant alleles, with no selectable marker, as described above.

Chimeras, generated by injection of a correctly reverted clone into blastocysts, were mated with C57BL/6 mice, and F1 heterozygotes were crossed to yield F2 offspring. Tail DNA was amplified by PCR, followed by ASO probe dot-blot hybridisation to identify homozygous mutants, as described above.

### RNA isolation and reverse transcription-PCR

Total RNA was isolated from WT and homozygous mutant retinas using the TRIzol Reagent (Invitrogen) and the RNeasy Mini Kit (Qiagen). Reverse transcription-PCR reactions were performed using the PrimeScript RT reagent Kit (Takara).

### Lysis of mouse tissues

A whole retina isolated from each mouse eye was disrupted and lysed by repetitive pipetting in 50 μL of RIPA buffer (10 mM Tris, pH 7.2, 0.15 mM NaCl, 1% Triton X-100, 1% sodium deoxycholate and 0.1% sodium dodecyl sulphate) containing 1:100 dilutions of phosphatase inhibitor cocktail (Nacalai Tesque) and protease inhibitor cocktail (Sigma) at 4 °C for 30 min. Insoluble material was removed from the homogenate by centrifugation at 20,000 × *g* for 40 min at 4 °C. Whole brains were homogenised as described^64^. The homogenate was lysed in RIPA buffer containing phosphatase and protease inhibitor cocktails and then centrifuged, as described above.

### Immunoblotting

Immunoblotting was performed essentially as described previously^63^. Briefly, proteins (25 µg per sample) were resolved on 8% sodium dodecyl sulphate polyacrylamide gel electrophoresis (SDS-PAGE) gels and electroblotted onto Hybond-ECL nitrocellulose membranes (Amersham). After blocking with 5% nonfat dry milk in Tris-buffered saline plus 0.05% Tween-20 (TBST), the membranes were incubated with a 1:2,000 dilution of mouse monoclonal anti-Src antibody clone 327 and a 1:2,000 dilution of rabbit monoclonal anti–β-tubulin (9F3) horseradish peroxidase (HRP)-conjugated antibody (Cell Signaling Technology) in TBST plus 2% nonfat dry milk, and then incubated with HRP-conjugated anti-mouse secondary antibody (1:4,000 dilution, GE Healthcare Life Sciences). The blots were visualised using ECL Plus western blotting detection reagents (Amersham). Images were acquired and quantitated using a LAS 1000 Image Analyzer (Fuji). Only values falling within the linear range were used for quantitative analysis.

### Immunoprecipitation and kinase assays

Whole brain tissue lysates (about 1 mg of protein) containing nearly equal levels of Src protein were treated with 0.5 μg of normal mouse IgG and 20 μL of protein A/G agarose (Santa Cruz Biotechnology) for 30 min at 4 °C and then centrifuged. The supernatants were incubated with 3 μL of mouse monoclonal anti-Src antibody 327 for 2 h at 4 °C, and then precipitated with 25 μL of protein A/G agarose for 1.5 h at 4 °C. The immunoprecipitates were washed three times with RIPA buffer, once with buffer containing 10 mM Tris-HCl (pH 7.0) and 0.15 M NaCl, and once with kination buffer (20 mM Tris, pH 7.0, 10 mM MnCl_2_), and then subjected to immunoblot analysis as described above. Autophosphorylation assays were performed as previously described^63^ with minor modifications. In brief, the washed immunoprecipitates were incubated for 20 min at 30 °C in 40 μL of kination buffer containing 5 μCi of [γ-^32^P]ATP and 1 μM ATP. The reaction was stopped with RIPA buffer, and proteins were resolved by 8% SDS-PAGE. Autophosphorylation was detected using a BAS 2500 Image Analyzer (Fuji).

### Detection of Src Ser75 phosphorylation

Src was immunoprecipitated with anti-Src antibody 327 from tissue lysate proteins (1 mg each), as described above. Immunoprecipitates were subjected to immunoblot analysis with an anti-Src (phospho-Ser75) antibody (1:750 dilution, Abcam). Blots were stripped and reprobed with the anti-Src antibody as described above.

### Histological examination of mutant mouse retina

Eyes were removed from WT and mutant mice (aged 3–23 months) and then fixed in 4% paraformaldehyde in phosphate buffer (pH 7.4). The samples were embedded in paraffin, and 3 μm sections were cut through the optic nerve. The sections were deparaffinised in xylene and rehydrated in a series of graded ethanol solutions. After haematoxylin and eosin staining, the processed retinal sections were analysed using a light microscope (Olympus) at a magnification of 10 × or 20 × by an examiner who was blinded to all information regarding the samples.

### Quantitation of RGCs

An eye was enucleated from each WT and mutant mouse (aged 3–23 months) and processed as a flattened whole mount using a published method^65^. Whole retinas were washed with PBS three times (5 min each) and permeabilised in 0.5% Triton-X 100 in PBS (0.1 M) for 15 min at room temperature. Retinas were washed three times with PBS and incubated with a mouse anti-Brn3a monoclonal antibody (1:250 dilution; MAB1585 clone 5A3.2; Millipore,) in blocking buffer (2% bovine serum albumin, 2% TritonX-100) at 4 °C overnight. Retinas were washed with PBS three times and incubated with a rabbit anti-mouse IgG secondary antibody conjugated to Alexa Fluor 568 (1:200 dilution, A-11061, Molecular Probes) for 3 h at room temperature. Retinas were washed three times with PBS and mounted onto slides with Fluorescent Mounting Medium (DAKO). Brn3a-positive RGCs were assessed by observing whole retinas under an epifluorescence microscope (BX50, Olympus) at a magnification of 20 × , and digitised images were compiled into a whole retina.

The number of Brn3a-positive RGCs was counted at two regions, namely, the mid-peripheral and peripheral regions of the retina, by an examiner who was blinded to all information regarding the samples. In each retina, the distance between the retinal margin and the optic nerve head was divided into thirds (Fig. 2b). The number of Brn3a-positive RGCs in the four boxed areas of equal size (400 × 400 μm) located at an equal distance of one-third from the optic nerve head (mid-periphery region) and at an equal distance of two-thirds from the optic nerve head (periphery region) were determined manually using the cell counter in ImageJ (Fig. 2b,c). The average value of the four boxed areas was used as the RGC number in the mid-periphery or periphery region of the retina of each mouse.

For investigations of the effect of ageing, samples from WT and mutant mice were categorised into two subgroups by age: young (3–6 months old) and ageing (16–23 months old).

### ROCK activity assay

A whole retina isolated from each mouse eye was disrupted and lysed by repetitive pipetting in lysis buffer (20 mM Tris, pH 7.4, 1% Triton X-100), containing 1:100 dilutions of phosphatase inhibitor cocktail 2 (Sigma) and protease inhibitor cocktail (Sigma) at 4 °C for 15 min. Insoluble material was removed from the lysate by centrifugation at 20,000 × *g* for 20 min at 4 °C. The protein concentration of the lysate was determined with a Bradford protein assay (Bio-Rad). The immunoassay for ROCK activity was conducted in duplicate according to the manufacturer’s instructions (CycLex) using equal amounts of protein (3 μg). For visualisation, the HRP substrate reagent was added to the wells and incubated for 15 min at room temperature. The absorbance was measured using a spectrophotometric microplate reader (SpectraMAX 340, Molecular Devices) at a single wavelength of 450 nm. The activity level (mU per mg of retina protein) was calculated using recombinant Rho-kinase II (CycLex) as a standard. The activity was corrected by subtracting the value in the presence of an inhibitor of Rho-associated kinases, Y-27632 (Cat. No., 688001, Millipore), from that in the absence of the inhibitor.

For ROCK immunoblotting, whole retinal proteins (10 μg per sample) were resolved on 4–15% SDS-PAGE gels and transferred via the Trans-Blot Turbo blotting system (Bio-Rad Laboratories) to the 0.2 μm polyvinylidene difluoride membranes supplied in the Trans-Blot transfer packs. The filter was separated into two pieces, one containing ROCK proteins and the other containing β-tubulin. ROCK filters were incubated with an anti-ROCK-1/2 rabbit polyclonal antibody (1:600 dilution, Cat. No., 07–1458, Millipore) for 16 h at 4 °C and then incubated with a HRP-conjugated anti-rabbit (1:5,000 dilution, GE Healthcare Life Sciences) secondary antibody. The β-tubulin filters were incubated with a rabbit monoclonal anti–β-tubulin (1:2,000 dilution, 9F3) HRP-conjugated antibody for 16 h at 4 °C. The blots were visualised using ECL Prime western blotting detection reagents. Images were acquired and quantitated using a LAS 4000 Image Analyzer (GE Healthcare Life Sciences). Only values falling within the linear range were used for quantitative analysis. ROCK protein levels in each retina were normalised to those of β-tubulin.

### IOP measurement

The IOP was measured in ketamine/xylazine-anesthetised mice between 14:00 h and 17:00 h with a rebound tonometer (TonoLab). The TonoLab tonometer provides a reliability score for each measured IOP value; according to the manufacturer’s recommendation, we used IOP values with good reliability. The IOP measurements were repeated until three reliable measurements were obtained, and the median of those three measurements was used as the IOP value for that set of measurements in each eye. The higher value of the right and left eyes was used as the IOP value for each mouse.

### Statistical analysis

The results are presented as means ± s.d. and were analysed by unpaired two-tailed *t*-test, factorial ANOVA followed by Bonferroni post hoc tests or paired factorial ANOVA followed by Bonferroni post hoc tests, as appropriate. All statistical analyses were performed using Ekuseru–Toukei 2012 software (Social Survey Research Information). The *P* values <0.05 were considered statistically significant.

### Data Availability

The datasets generated during and/or analysed during the current study are available from the corresponding author upon reasonable request.

## Electronic supplementary material

Supplementary Information
