# Supplementary material for: A Ser75-to-Asp phospho-mimicking mutation in Src accelerates ageing-related loss of retinal ganglion cells in mice
Source: Sci Rep. 2017 Dec 1;7:16779. doi: 10.1038/s41598-017-16872-7 (PMC5711949; doi:10.1038/s41598-017-16872-7)
Supplement: Supplementary file 1 — Supplementary Information [file 41598_2017_16872_MOESM1_ESM.pdf]

Supplementary Information

A Ser75-to-Asp phospho-mimicking mutation in Src accelerates ageing-related loss of retinal ganglion cells in mice

Kenji Kashiwagi[1‡], Sadahiro Ito[2†], Shuichiro Maeda[2] and Goro Kato[2*‡]

[1]Department of Ophthalmology, [2]Department of Biochemistry, Faculty of Medicine, Graduate Faculty of Interdisciplinary Research, University of Yamanashi, Yamanashi 409-3898, Japan.

[†]Present address: Center for Life Science Research, University of Yamanashi, 1110 Shimokato, Chuo, Yamanashi 409-3898, Japan.

[‡]K.K. and G.K. contributed equally to this work.

*Correspondence should be addressed to Goro Kato, Department of Biochemistry, Faculty of Medicine, Graduate Faculty of Interdisciplinary Research, University of Yamanashi, 1110 Shimokato, Chuo, Yamanashi 409-3898, Japan.

Email: gkato@yamanashi.ac.jp

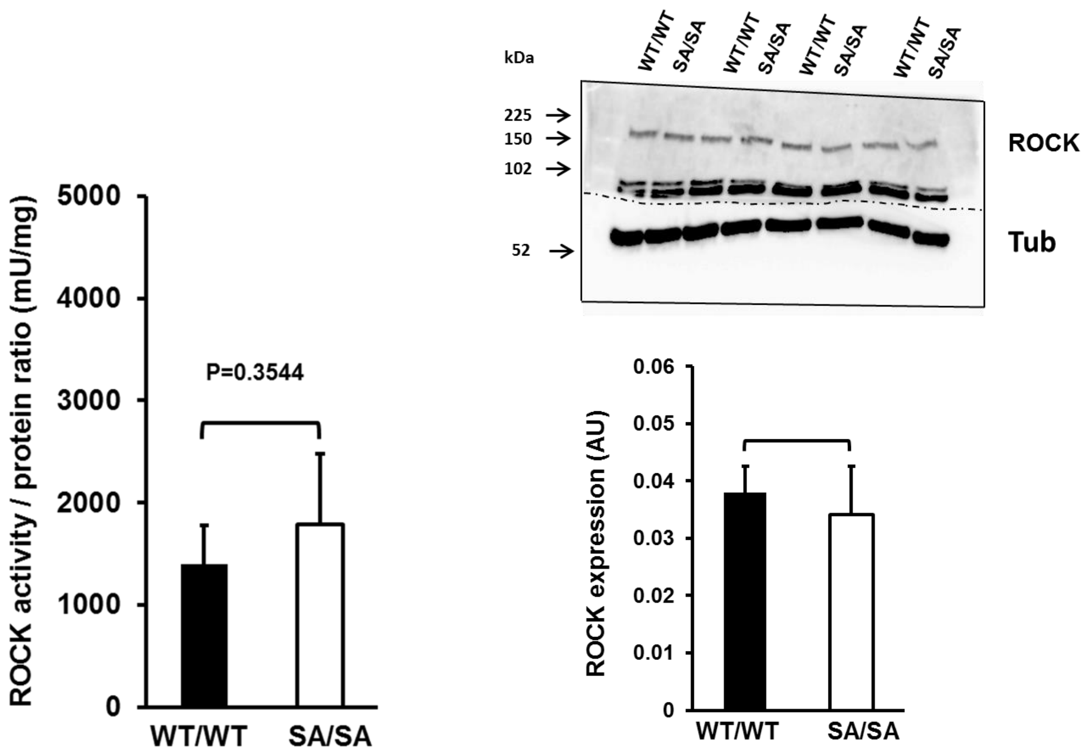

Supplementary Figure S1.

ROCK activity/protein ratios in retinas obtained from young SA mutant mice. The lysate of each retina (10 μg) was subjected to anti-ROCK immunoblot analysis (**right**, upper panel). ROCK protein levels were normalised to those of 55 kDa β-tubulin (Tub) (**right**, lower panel) and used to estimate the specific kinase activity (**left**). n = 4. Data are means ± s.d. Unpaired two-tailed *t*-test.

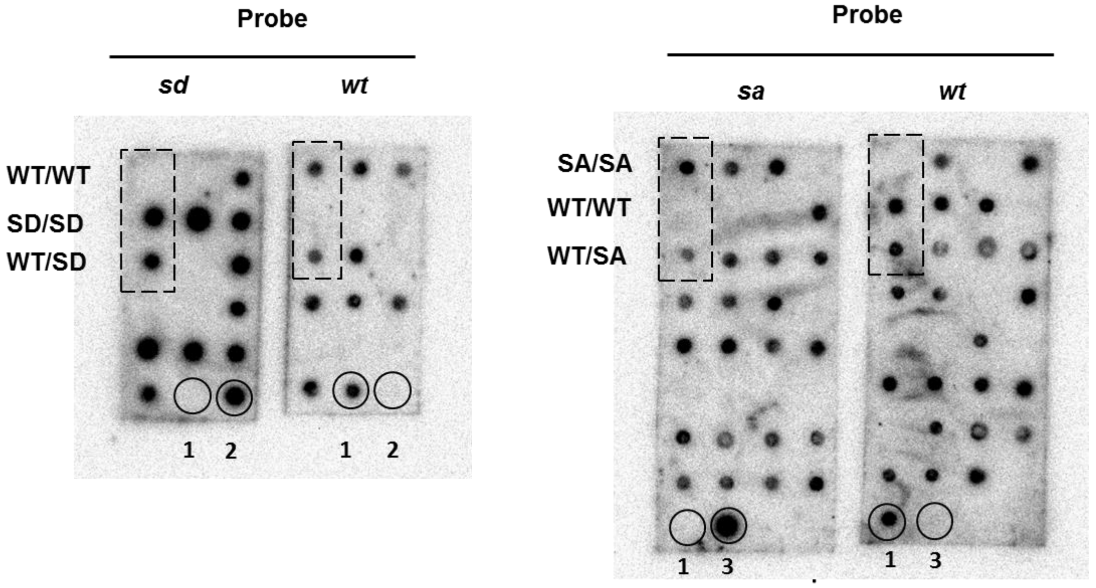

Supplementary Figure S2.

The full-size dot blots for Figure 1a. Dashed line boxes indicate the cropped images used in Fig. 1a. PCR products from littermates derived from SD or SA heterozygotes were spotted onto duplicate membranes, denatured, neutralized, and fixed. One membrane was hybridized with ^{32}P-labelled ASO probe *wt,* and the other was hybridized with ^{32}P-labelled ASO probe *sd* (left) or *sa* (right) to detect the mutant allele .Circle 1, cloned genomic c-*src* DNA fragment (wild-type); circle 2, cloned genomic c-*src* DNA fragment bearing SD mutation; circle 3, cloned genomic c-*src* DNA fragment bearing SA mutation.

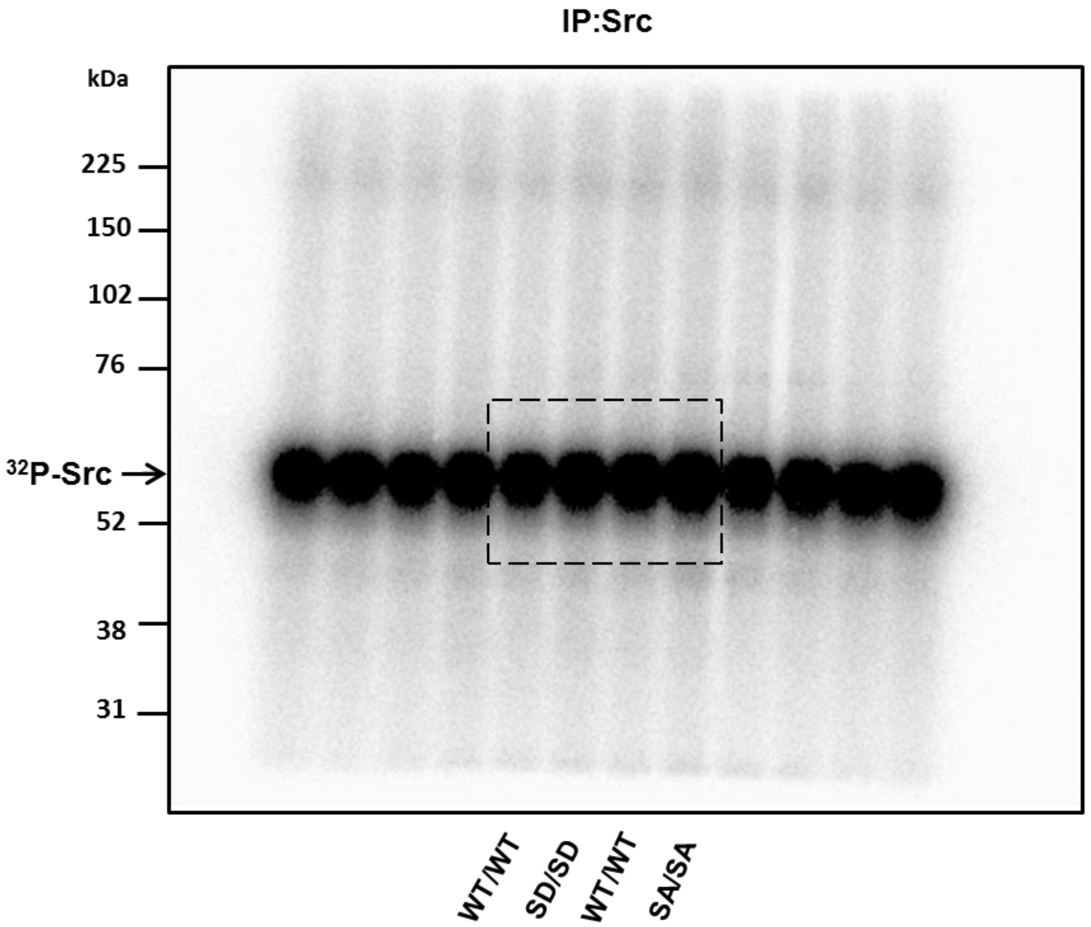

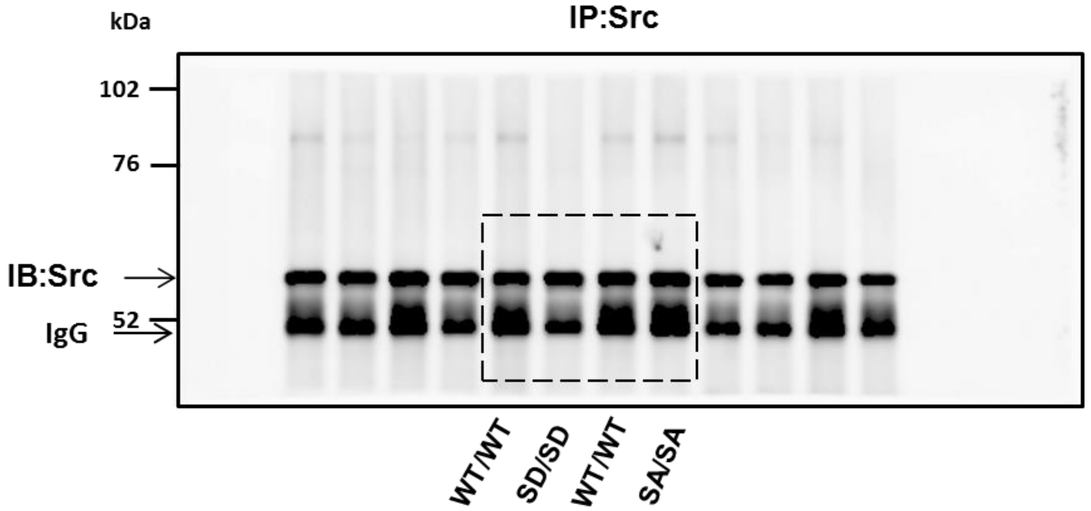

Supplementary Figure S3.

The full-length blot (lower) and autoradiograph (upper) for Figure 1c. Dashed line boxes indicate the cropped images used in Fig. 1c.

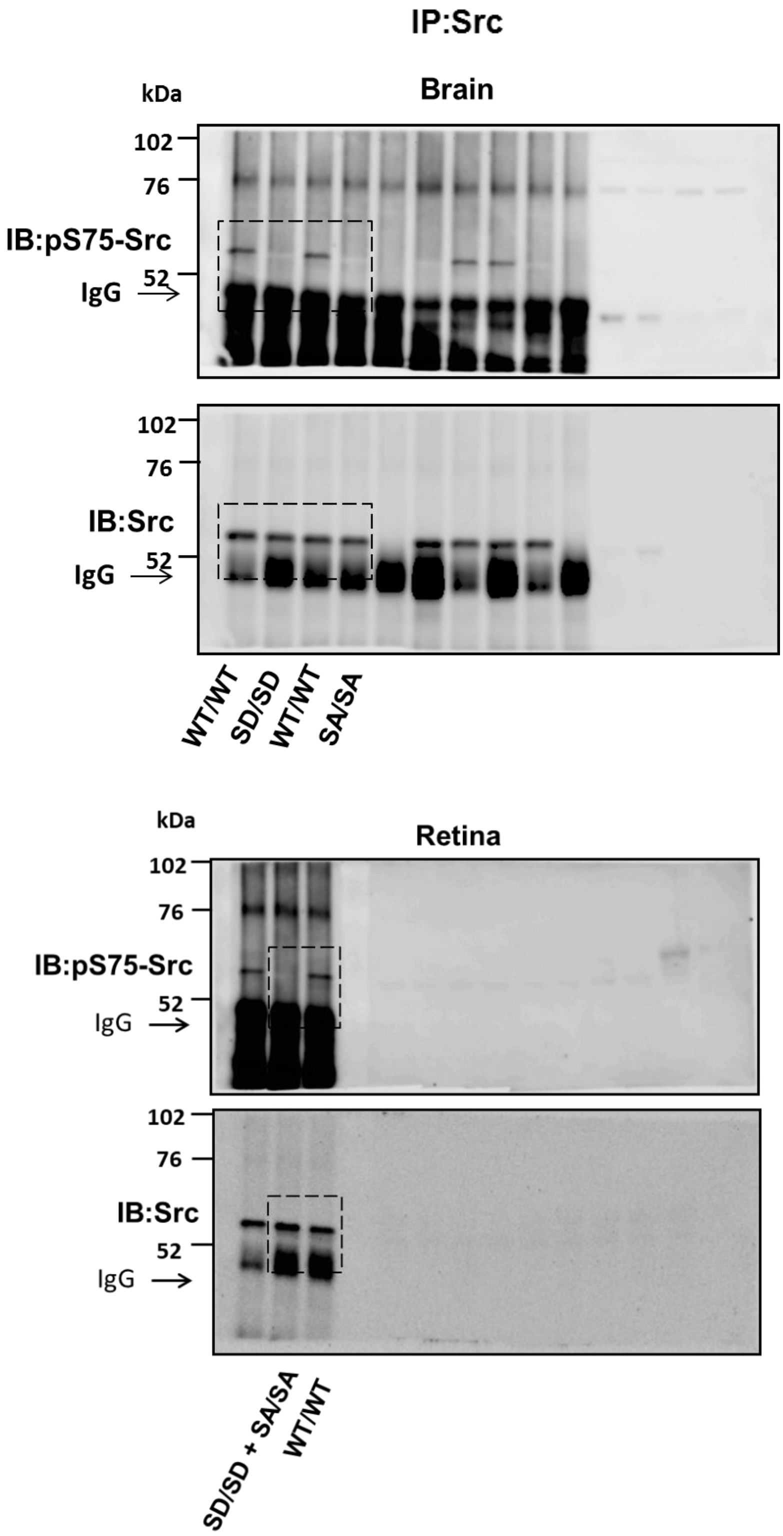

Supplementary Figure S4.

The full-length blots for Figure 1d. Dashed line boxes indicate the cropped images used in Fig. 1d.

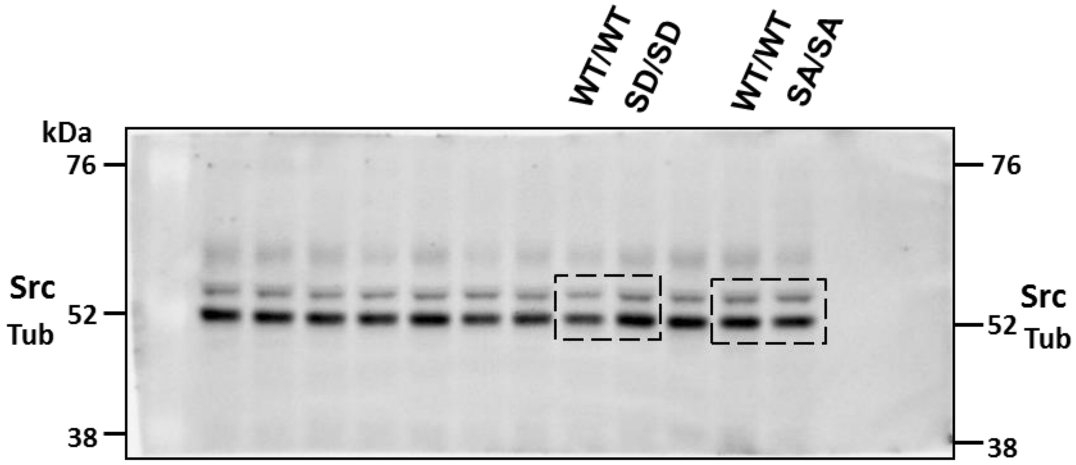

Supplementary Figure S5.

The full-lengh blot for Figure 1e. Dashed line boxes indicate the cropped images used in Fig. 1e.

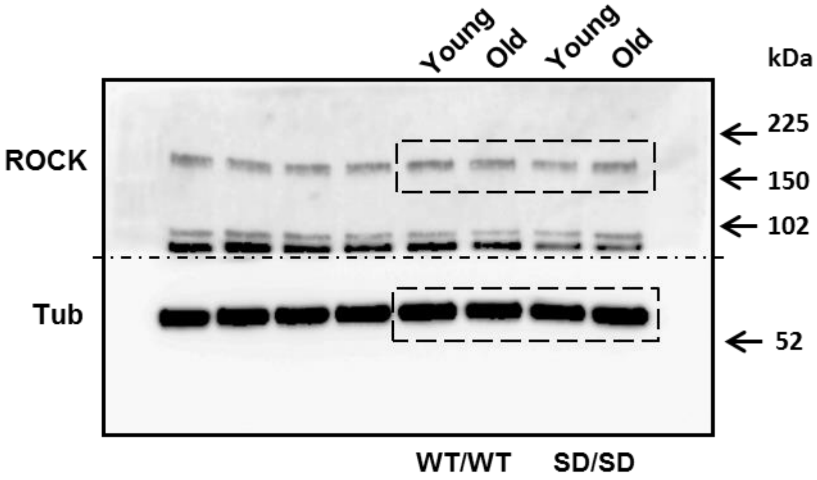

Supplementary Figure S6.

The full-lengh blot for Figure 3c. Dashed line boxes indicate the cropped images used in Fig. 3c. The filter was separated along one dot chain line into two pieces, one containing ROCK proteins and the other containing β-tubulin.
